# Lower extremity lymphedema in patients with gynecologic cancer: Validation of the Gynecologic Cancer Lymphedema Questionnaire (GCLQ) in German language and investigation of lymphedema real-world treatment

**DOI:** 10.1007/s00404-024-07886-4

**Published:** 2024-12-23

**Authors:** Henrike Meyer, Andreas Hinz, Christiane Weisgerber, Adrian Pilny, Nadja Dornhöfer, Anja Mehnert-Theuerkauf, Bahriye Aktas, Benjamin Wolf

**Affiliations:** 1 Department of Gynecology, University Hospital Leipzig, Liebigstraße 20a, 04103 Leipzig, Germany; 2 Department of Medical Psychology and Medical Sociology, Comprehensive Cancer Center Central Germany (CCCG), University Medical Center Leipzig, Leipzig, Germany; 3 Edwin L. Steele Laboratories, Department of Radiation Oncology, Massachusetts General Hospital, Boston, USA

**Keywords:** GCLQ-GER, Clinical screening tool, Patient-reported outcome measures (PROMs), Secondary prevention of lymphedema, Quality of life

## Abstract

**Purpose:**

The Gynecologic Cancer Lymphedema Questionnaire (GCLQ) is an established patient-reported outcome measure for lower extremity lymphedema (LEL) in gynecologic oncology. We aimed to validate the GCLQ in German language (GCLQ-GER) for lymphedema detection in German-speaking patients and also investigated real-world patterns of lymphedema treatment.

**Methods:**

The GCLQ was translated from English into German in accordance with the standards of a professional translation process. Subsequently, the questionnaire was administered in a prospective observational study including 102 patients who had undergone lymph node dissection (LND) within gynecologic cancer surgery. Various test quality criteria were calculated for the GCLQ-GER. As gold standard of testing methods, patients were clinically evaluated for LEL, and limb volume measurements were taken. Further data for lymphedema treatment were collected in patients with lymphedema diagnosis.

**Results:**

Patients with LEL had increased GCLQ-GER total scores (mean 7.27) compared to patients without LEL (mean 1.81), p < 0.001. High diagnostic accuracy was indicated by the large area under the receiver operating characteristics curve (AUC) of 0.874 (95% CI 0.802–0.946). Based on sensitivity and specificity values ≥ 79.0%, the GCLQ total score ≥ 4 was determined as the optimal cut-off value to identify LEL. Excellent internal consistency was demonstrated by Cronbach’s alpha of 0.876. The clinical examination revealed a LEL prevalence of 48.0% (n = 49), and 85.7% (n = 42) of these patients received treatment.

**Conclusion:**

The GCLQ-GER is a valid and feasible patient-reported outcome measure for lymphedema detection in German-speaking gynecologic cancer survivors. Its clinical introduction could improve secondary prevention of lymphedema and real-world treatment.

**Supplementary Information:**

The online version contains supplementary material available at 10.1007/s00404-024-07886-4.

## What does this study add to the clinical work

**Table:** 

Secondary lower extremity lymphedema after gynecologic cancer surgery is associated with physical and psychosocial burden and impact on the health-related quality of life. This study validated the Gynecologic Cancer Lymphedema Questionnaire in German language (GCQL-GER) and paved the way to broadly introduce this patient-reported screening tool for lymphedema in clinical practice.	

## Introduction

Lymphedema of the lower extremities (LEL) represents a serious complication of surgical cancer therapy in gynecologic oncology. Incidence rates of up to 70% demonstrate the high relevance of secondary lymphedema after lymph node dissection (LND) in cancer therapy [1–3]. Lymphedema affects physical and psychosocial aspects of quality of life [4–7] and appears in numerous patients within the first few weeks after surgery [1, 8]. Symptoms commonly consist of swelling with fluid accumulation, numbness or firmness of the skin, a feeling of heaviness and significant limitation of movement and more severely affected patients [9]. Physical impairments often lead to functional disorders including limited limb mobility in daily activities, reduced walking distance and changes in choice of clothing and shoes [5]. From a psychosocial perspective, lymphedema is associated with a negative perception of body image, anxiety about appearance and fatigue symptoms [4, 10]. Carter et al. reported that patients are constantly reminded of their cancer experience by lymphedema symptoms, leading to chronic mental distress and challenging disease coping [4]. Moreover, lymphedema therapy accompanies patients for years. Time expenditures and treatment costs for compression therapy and lymphatic drainage directly affect patients’ resources and finances [11, 12]. For those reasons, lymphedema causes an additional burden of disease for gynecologic cancer patients.

The pathomechanism of secondary lymphedema is based on the interruption of pelvic and inguinal lymphatic pathways by LND [13–15]. Insufficient drainage of lymphatic fluid contributes to interstitial fluid accumulation along the osmotic gradient, resulting in edema. Chronic interstitial inflammation with monocyte and fibroblast infiltration leads to thickening of the cutis and subcutis, transitioning into fibrosis and sclerosis. Clinically, lymphedema can be divided into four consecutive stages [13]. Recognizable lymphedema is often preceded by a latent stage (0) of subclinical symptoms. Stage 1 is fully reversible during leg raising, whereas stages 2 and 3 are characterized by position-independent irreversible swelling, skin induration and fibrosis. In advanced stages, local immunodeficiency facilitates the development of trophic changes and chronic wounds [16].

The necessity for earliest possible lymphedema detection to facilitate secondary prevention after surgical intervention remains undisputable. Lymphedema diagnosis is commonly based on clinical examination while more cumbersome quantitative methods such as multiple measurements of limb circumferences, water displacement and bioimpedance spectroscopy are usually performed in the context of clinical studies only [17, 18]. Importantly, the correlation between the extent of objectively quantified lymphedema and subjective disease burden varies. Therefore, the integration of subjective patient-reported outcome measures (PROMs) has become increasingly important [3]. To facilitate easier and more comprehensive lymphedema assessment in gynecologic cancer patients, the gynecologic cancer lymphedema questionnaire (GCLQ) was developed by Lockwood et al., modeled after the Lymphedema Breast Cancer Questionnaire (LBCQ) for upper-extremity lymphedema assessment after breast cancer surgery [19]. The questionnaire was validated in 2010, initially in English language by Carter et al. [20]. Subsequently, the GCLQ has been translated and validated in Korean, Norwegian and Turkish language [21–24]. Several landmark studies on lymphedema in gynecologic cancer patients have since been published. We have previously explored the usefulness of a preliminary, non-validated version of the GCLQ in German language in vulvar cancer patients [25], however, a validated German version of the GCLQ is not yet available [6]. Here, we realize the initial validation of the GCLQ in German language (GCLQ-GER), catering to the largest native language group in Europe [26].

## Methods

### Study design and participants

This prospective monocentric observational study involved 112 gynecologic cancer patients who were treated in the Department of Gynecology of the University Hospital Leipzig in Germany. Data were collected as part of our cancer aftercare program in the outpatient center. Patients meeting the following inclusion criteria were eligible for enrollment: (1) diagnosis of gynecologic cancer, (2) LND as part of surgical cancer therapy, (3) minimum age 18 years, (4) informed consent for study participation. Exclusion criteria were: (1) cardiac and renal diseases resulting in edema, (2) surgical intervention without dissection of at least one lymph node, (3) language barrier for non-German native speakers, (4) lack of cognitive or mental ability to give consent, (5) age under 18 years. Medical history data was collected from our hospital database. The study was approved by the Ethics Committee of Leipzig University (Number 261/21-ek).

### Clinical diagnosis of lymphedema and limb volume measurements

To assess lymphedema prevalence in this study cohort, patients were clinically examined for signs of lymphedema by experienced gynecologic oncologists. This clinical assessment represents the gold standard of testing methods in this study to which the assessment by GCLQ-GER is compared.

The circumference measurement method was applied for total limb volume measures to augment the clinical diagnosis. Patients were placed on an examination table and multiple limb circumference measurements were taken on both limbs from the ankle to the inguinal fold in 10 cm intervals. As described by Podleska et al., total limb volumes were calculated to finally determine the limb volume for each extremity [27].

### Translation and validation of the GCLQ-GER

The GCLQ consists of 20 items which are each scored in a binary fashion (i.e. 0 or 1), yielding a maximum score of 20. All questions refer to the past 4 weeks (supplementary Table S1). The following symptom clusters were applied for ranking of clinical relevance: (1) swelling: items 8, 9, 20; (2) numbness: items 7, 12, 15, 16; (3) heaviness: item 14; (4) aching: item 17; (5) swelling limb: items 18, 19; (6) infection: items 10, 11, 13, and (7) physical function: items 1–6 [20].

The original GCLQ in English was first translated into German by two independent investigators and then retranslated by a native English speaker. Minor linguistic modifications were made to ensure consistency in the final German translation. All patients completed the GCLQ-GER postoperatively during one of their aftercare visits in our outpatient center.

For testing the GCLQ-GER´s structural validity, the internal consistency was investigated on an item level using Cronbach's alpha. Moreover, the receiver operating characteristic (ROC) curve and area under the ROC curve (AUC) were calculated for the GCLQ-GER total and symptom cluster scores with a confidence interval of 95%. To identify the optimal GCLQ-GER-cut-off value, several cutoffs were determined and their corresponding testing statistics (i.e., sensitivity, specificity, positive predictive value [PPV], negative predictive value [NPV]) were calculated. The concordance between clinically diagnosed (true) lymphedema and lymphedema detected by GCLQ-GER was assessed using McNemar’s test and Cohen’s kappa statistic.

### Lymphedema treatment

To investigate real-world lymphedema treatment patterns, further data on previous or current therapy were gathered for patients with clinically diagnosed lymphedema. This assessment included a survey of applied treatment methods, the latency of treatment after surgery and treatment frequencies.

### Statistical analysis

Continuous variables are reported either as means with standard deviation or medians with interquartile range (IQR), while categorical variables are given as percentages. Non-normal distribution was assessed with the Shapiro-Wilk test and non-parametric statistical tests were used accordingly for correlation analysis and comparison of means. The chi-square test was used for contingency analysis. Statistical significance was defined as p ≤ 0.05. All analyses were performed using the statistical software SPSS (SPSS Statistics, Version 29.0.1.0; IBM Corp., NY, USA).

## Results

### Patient characteristics

Of the 112 patients who provided informed consent to participate in this study, 10 subjects were subsequently excluded because of missing data. Therefore, data from 102 patients were analyzed.

Results are summarized in Table 1. The most frequently registered cancer entities were cervical and ovarian cancer, each in 39 patients (38.2%), followed by endometrial (n = 14, 13.7%) and vulvar cancer (n = 10, 9.8%). Almost half of all patients had stage I disease (n = 48, 47.1%) according to the current staging protocols of the Fédération Internationale de Gynécologie et d'Obstétrique (FIGO). Radical hysterectomy with LND (n = 47, 46.1%) and cytoreductive surgery (n = 39, 38.2%) were the most frequently performed surgeries. Comorbidities did not differ significantly between patients with and without lymphedema. Arterial hypertension was identified as the most frequent comorbidity (n = 45, 44.1%), followed by a history of previous pelvic surgery or radiotherapy (n = 35, 34.3%).

**Table 1:** Patient characteristics

Characteristics	LEL	No LEL	Total	p-value*
Patients, n (%)	49 (48.0)	53 (52.0)	102 (100.0)	
Age (years)				
Mean (SD)	57.0 (12.38)	59.7 (11.0)	58.4 (11.7)	0.205
Median (IQR)	59.0 (49.0–66.0)	62.0 (54.0–68.0)	61.5 (52.0–67.0)	
Body mass index (kg/m^2^)				
Mean (SD)	26.2 (4.9)	27.3 (5.2)	26.8 (5.1)	0.227
Median (IQR)	25.2 (22.9–28.0)	26.3 (24.3–29.7)	25.7 (23.3–29.1)	
Tumor entity, n (%)				
Cervical cancer	26 (53.0)	13 (24.5)	39 (38.2)	0.003
Endometrial cancer	6 (12.2)	8 (15.1)	14 (13.7)	0.676
Vulvar Cancer	4 (8.2)	6 (11.3)	10 (9.8)	0.592
Ovarian Cancer	13 (26.5)	26 (49.1)	39 (38.2)	0.019
FIGO stage, n (%)				
I	25 (51.0)	23 (43.4)	48 (47.1)	0.441
II	13 (26.5)	5 (9.4)	18 (17.6)	0.024
III	6 (12.2)	20 (37.8)	26 (25.5)	0.003
IV	5 (10.2)	5 (9.4)	10 (9.8)	0.896
Surgical intervention, n (%)				
Radical hysterectomy with LND	30 (61.2)	17 (32.1)	47 (46.1)	0.003
Radical vulvar surgery with LND	4 (8.2)	6 (11.3)	10 (9.8)	0.592
Cytoreductive surgery (tumor debulking)	12 (24.5)	27 (50.9)	39 (38.2)	0.006
Laparoscopic hysterectomy	3 (6.1)	3 (5.7)	6 (5.9)	0.921
Surgical site complications n (%)	9 (18.4)	14 (26.4)	23 (22.5)	0.331
LND, n (%)				
Pelvic	21 (42.9)	10 (18.9)	31 (30.4)	0.008
Pelvic and paraaortic	22 (44.9)	18 (34)	40 (39.2)	0.258
Inguinal	4 (8.2)	6 (11.3)	10 (9.8)	0.592
Sentinel LND	2 (4.0)	5 (9.4)	7 (6.8)	0.285
Other lymphatic drainage pathways	0 (0.0)	14 (26.4)	14 (13.7)	< 0.001
Number of dissected LN, n (%)				
Mean (SD)	37.9 (21.5)	22.3 (19.6)	29.6 (21.8)	< 0.001
Median (IQR)	36.5 (22.5–54.2)	17.0 (4.0–37.7)	27.5 (9.5–44.0)	
1–4	3 (6.1)	14 (26.4)	17 (16.7)	0.006
5–19	7 (14.3)	15 (28.3)	22 (21.6)	0.086
20–39	18 (36.7)	12 (22.6)	30 (29.4)	0.119
40–60	12 (24.5)	9 (17.0)	21 (20.6)	0.349
> 60	9 (18.4)	3 (5.7)	12 (11.8)	0.047
Adjuvant treatment, n (%)				
Radiotherapy	3 (6.1)	0 (0.0)	3 (2.9)	0.067
Chemotherapy	19 (38.8)	24 (45.3)	43 (42.1)	0.506
Comorbidities, n (%)				
Hypertension	18 (36.7)	27 (50.9)	45 (44.1)	0.149
Obesity	10 (20.4)	13 (24.5)	23 (22.5)	0.619
Previous pelvic surgery/ radiation	17 (34.7)	18 (34.0)	35 (34.3)	0.938
Diabetes mellitus	6 (12.2)	8 (15.1)	14 (13.7)	0.676
Smoking	8 (16.3)	8 (15.1)	16 (15.7)	0.864
Depression	1 (2.0)	4 (7.5)	5 (4.9)	0.198
Previous malignancies	5 (10.2)	7 (13.2)	12 (11.8)	0.638
Varices	5 (10.2)	2 (3.8)	7 (6.8)	0.199

*LEL* lower extremity lymphedema, *SD* standard deviation, *IQR* interquartile range, *FIGO* International Federation of Gynecology and Obstetrics, *LND* lymph node dissection, *LN* lymph nodes

*p-values for the characteristics patient´s age, body mass index and the number of dissected LN were determined by using the Mann–Whitney-U-Test for independent samples with non-normal distribution. All other p-values were calculated with the Chi-square test

### Lymphedema prevalence

**Fig. 1:**
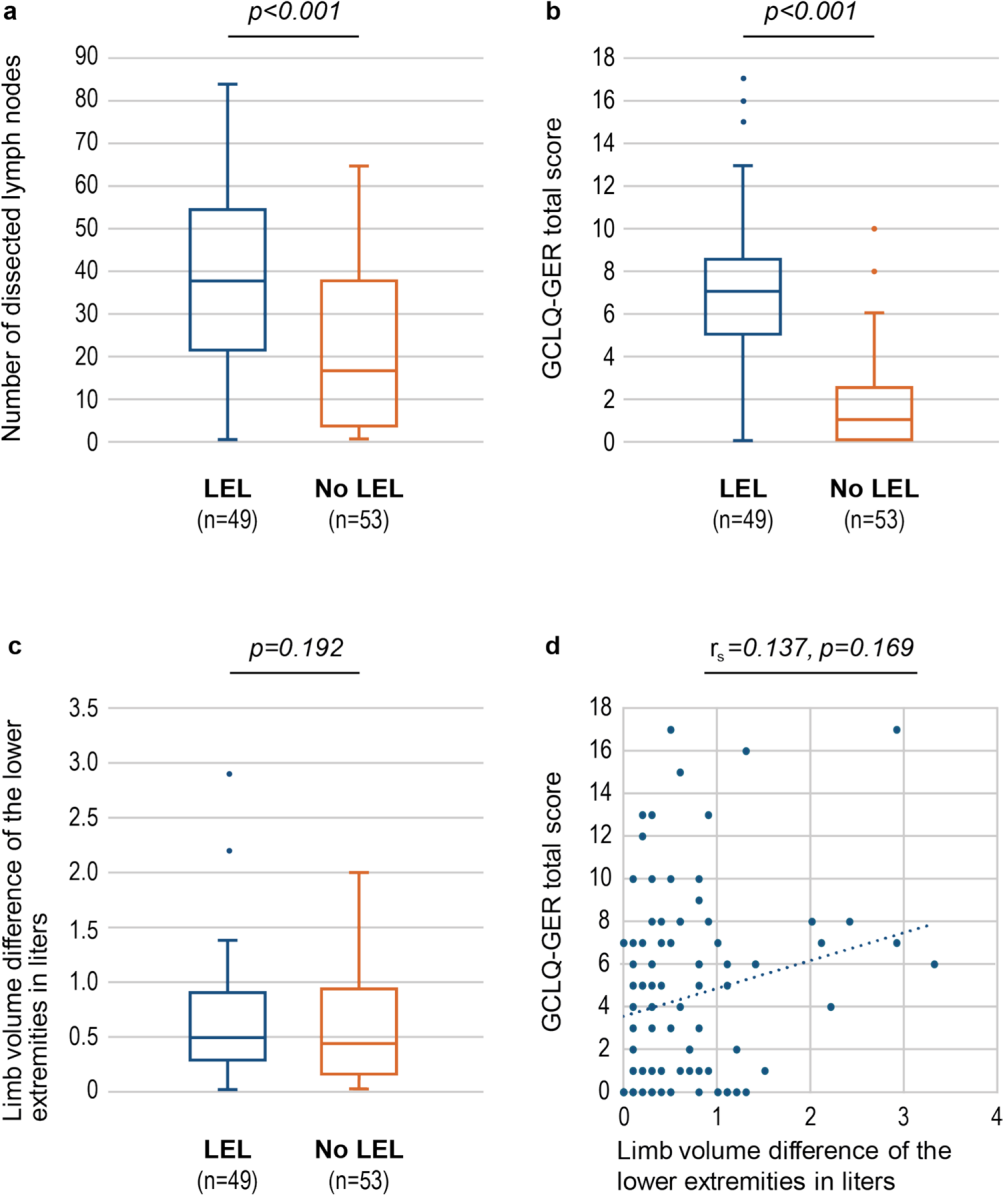
Correlation of clinically diagnosed lymphedema with (**a**) number of dissected lymph nodes, **b** GCLQ-GER total score, **c** limb volume difference of the lower extremities in liters and **d** correlation of limb volume difference of the lower extremities in liters with the GCLQ-GER total score. LEL, lower extremity lymphedema; No LEL, no presence of lower extremity lymphedema. *p* values for (**a**), (**b**) and (**c**) were determined by using Mann–Whitney-U-Test. Correlation analysis (**d**) was performed by using Spearman´s correlation coefficient r_s_

Clinical assessment revealed a lymphedema prevalence of 48.0% (n = 49), shown in Table 1. Among patients with lymphedema, cervical cancer (n = 26, 53.0%) and ovarian cancer (n = 13, 26.5%) were found to be the most frequent cancer entities. Radical hysterectomy with LND (n = 30, 61.2%) was the most common surgical intervention in lymphedema patients, with significant difference compared to patients not affected by lymphedema (p = 0.003). Furthermore, the number of dissected lymph nodes was significantly higher in patients with lymphedema (median 36.5, IQR 22.5–54.2) in comparison to patients without (median 17.0, IQR 4.0–37.7, p < 0.001, Fig. 1a).

### Patient-reported lymphedema on the GCLQ-GER

**Fig. 2:**
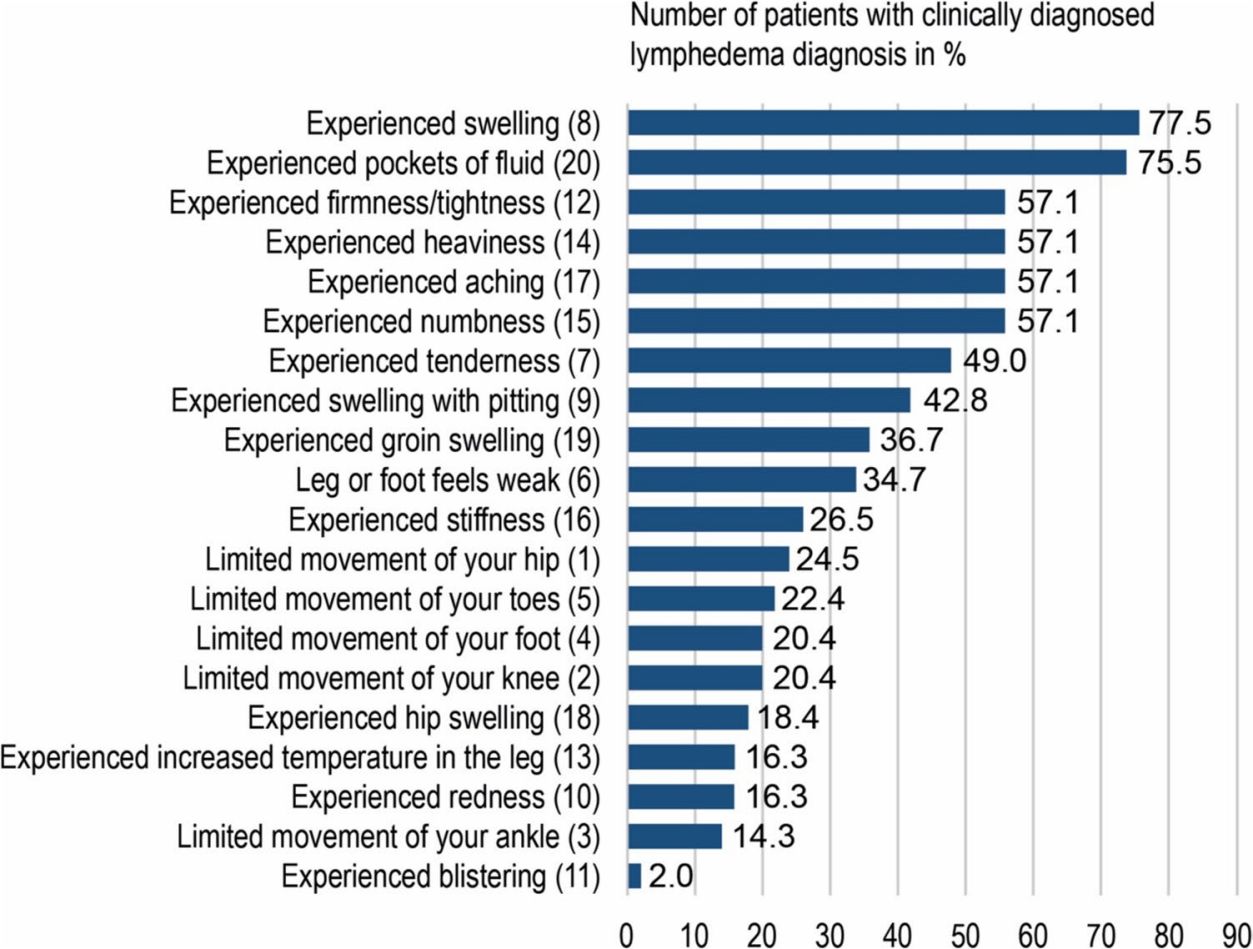
Frequency of patient-reported lymphedema symptoms on the GCLQ-GER in patients with clinically diagnosed lymphedema

Mean GCLQ-GER scores were significantly higher in patients with lymphedema compared to patients without it (7.27 vs. 1.81, p < 0.001, Fig. 1b). Lymphedema as assessed by GCLQ-GER measures was found in 52.0% (n = 53) using a cut-off value ≥ 4. This cut-off value was determined to distinguish between patients with and without lymphedema with similar accuracy as the gold standard of a physician-objectified diagnosis. GCLQ-GER-cut-off values with calculated test quality criteria are demonstrated in Table 2 and supplementary Figure S2. Patients´ perception revealed the following decreasing order of lymphedema-associated symptoms: swelling (n = 38, 77.5%), pockets of fluid (n = 37, 75.5%), firmness (n = 28, 57.2%) and heaviness (n = 28, 57.2%). Blistering (n = 1, 2%) and limited movement of ankle (n = 7, 14.3%) were reported significantly less frequently (Fig. 2 and supplementary Table S3).

**Table 2:** Potential clinical GCLQ-GER-cut-off values for patient-reported lymphedema assessment in clinical practice

Cut-offs*	≥ 2	≥ 3	≥ 4	≥ 5	≥ 6
Patient-reported lymphedema on the GCLQ-GER, n (%)	62 (60.8)	57 (55.9)	53 (52.0)	49 (48.0)	42 (41.2)
Sensitivity, %	95.9	89.8	85.7	77.6	73.5
Specificity, %	71.7	75.5	79.2	79.2	88.7
Positive predictive value (PPV), %	75.8	77.2	79.2	77.5	85.7
Negative predictive value (NPV), %	95.0	88.9	85.7	79.3	78.4
Cohen´s Kappa	0.669	0.649	0.648	0.568	0.625
McNemar´s test	0.002	0.096	0.481	1.000	0.167

*All cut-offs were determined for the total number of 102 patients. Lymphedema was present in 48.0% (n = 49) of patients

### Validation of the GCLQ-GER

**Fig. 3:**
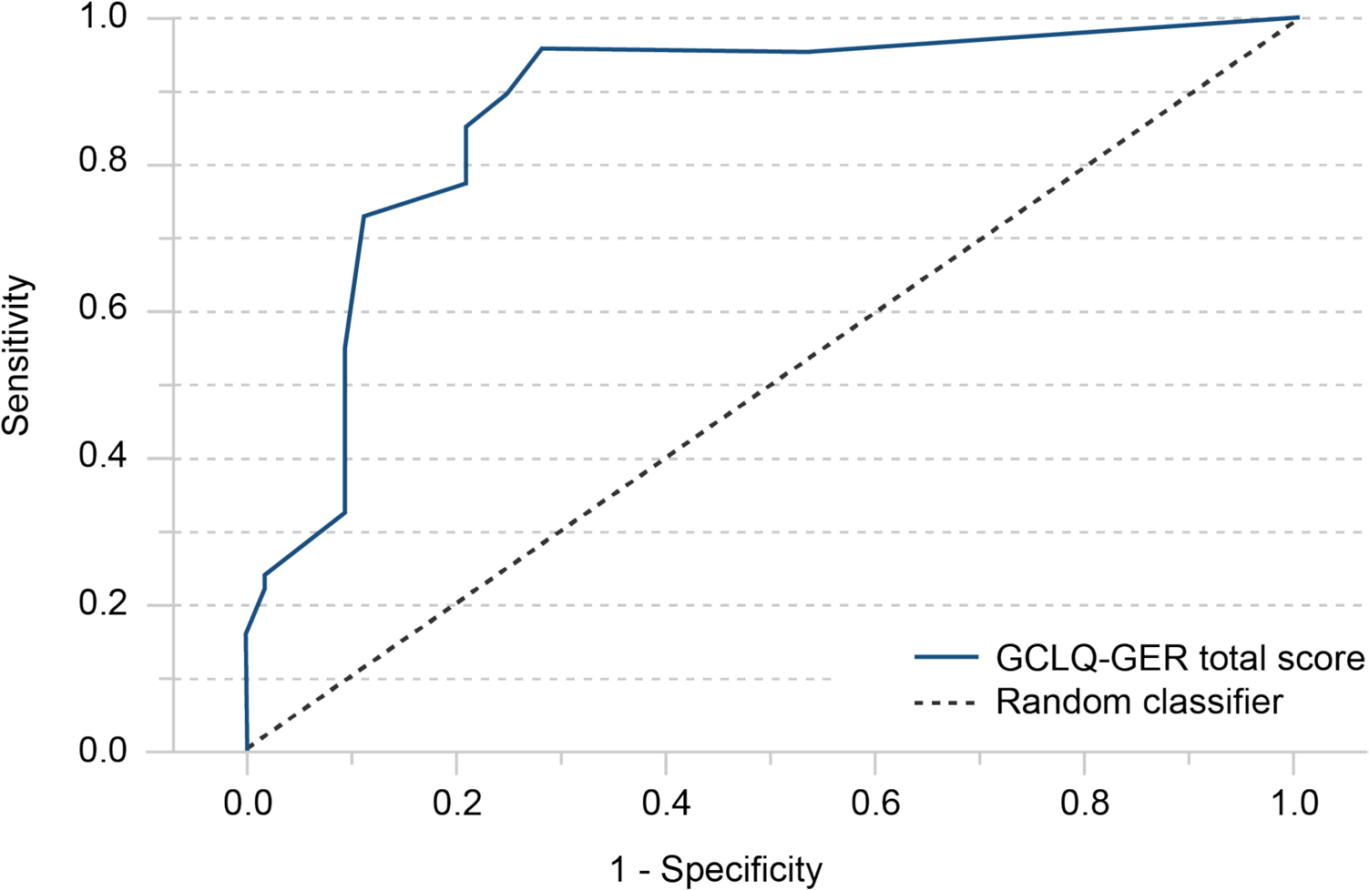
Receiver Operating Characteristic (ROC) curve for the patient-reported GCLQ-GER total score against the gold standard of clinically diagnosed lymphedema

The GER-GCLQ was validated for patient-based lymphedema assessment and compared against the gold standard of clinical examination. The validation was based on the ROC curve for the GCLQ-GER total and symptom cluster scores, plotted in Fig. 3 and shown in supplementary Table S4, respectively. The large AUC of 0.874 (95% confidence interval: 0.802–0.946), calculated for the GCLQ-GER total score, confirmed high diagnostic accuracy and measuring quality. Furthermore, all seven GCLQ-GER symptom clusters showed significantly higher mean scores when in patients with clinically diagnosed lymphedema (p < 0.05, Table 3). Especially symptom clusters 1 (swelling) and 2 (numbness) identified patients with clinically diagnosed lymphedema very effectively and were characterized by large AUCs, visualized in supplementary Table S4. For identifying lymphedema by using GCLQ-GER in clinical practice, five potential GCLQ-GER cut-off values (greater than or equal to 2, 3, 4, 5 or 6) were found to yield sensitivity and specificity greater than 79.0% in ROC analysis (Table 2). High diagnostic statistical accuracy as measured by PPV and NPV was calculated for each of these cut-off values. The cut-off value ≥ 4 performed most similar to the test gold standard, confirmed by high interrater reliability as Cohen´s kappa and McNemar´s test. Cronbach’s alpha of 0.876 indicated an excellent internal consistency for the GCLQ-GER.

**Table 3:** Patient-reported GCLQ-GER total score and GCLQ-GER symptom cluster scores for patients with lymphedema compared to patients without lymphedema

		LEL (n = 49)	No LEL (n = 53)	p-value*
GCLQ-GER total score	Mean, SD	7.27, 3.999	1.81, 2.711	< 0.001
	Median, IQR	7, 5.9–8.0	1, 0.0–2.0	
GCLQ-GER symptom cluster	Score, n (%)			
(1) Swelling: Items 8, 9, 20				
	0	7 (14.3)	45 (84.9)	< 0.001
	1	6 (12.3)	4 (7.5)	
	2	18 (36.7)	2 (3.8)	
	3	18 (36.7)	2 (3.8)	
(2) Numbness: Items 7, 12, 15, 16				
	0	6 (12.3)	36 (67.9)	< 0.001
	1	17 (34.7)	12 (22.7)	
	2	10 (20.4)	3 (5.6)	
	3	8 (16.3)	1 (1.9)	
	4	8 (16.3)	1 (1.9)	
(3) Heaviness: Item 14				
	0	21 (42.9)	45 (84.9)	< 0.001
	1	28 (57.1)	8 (15.1)	
(4) Aching: Item 17				
	0	21 (42.9)	39 (73.6)	0.002
	1	28 (57.1)	14 (26.4)	
(5) Swelling limb: Items 18, 19				
	0	29 (59.2)	49 (92.5)	< 0.001
	1	13 (26.5)	4 (7.5)	
	2	7 (14.3)	0	
(6) Infection: Items 10, 11, 13				
	0	37 (75.5)	50 (94.4)	0.006
	1	7 (14.3)	3 (5.6)	
	2	5 (10.2)	0	
	3	0	0	
(7) Physical function: Items 1–6				
	0	19 (38.8)	38 (71.7)	< 0.001
	1	14 (28.6)	9 (17)	
	2	7 (14.3)	3 (5.6)	
	3	3 (6.1)	0	
	4	3 (6.1)	2 (3.8)	
	5	0	1 (1.9)	
	6	3 (6.1)	0	

*LEL* lower extremity lymphedema, *SD* standard deviation

*p values for mean comparisons of the GCLQ-GER total score and GCLQ-GER cluster 1, 2, 5, 6 and 7 were determined by using the Mann–Whitney-U-Test, while p-values for GCLQ-GER cluster 3 and 4 were performed with the Chi-square test

### Lymphedema treatment

**Fig. 4:**
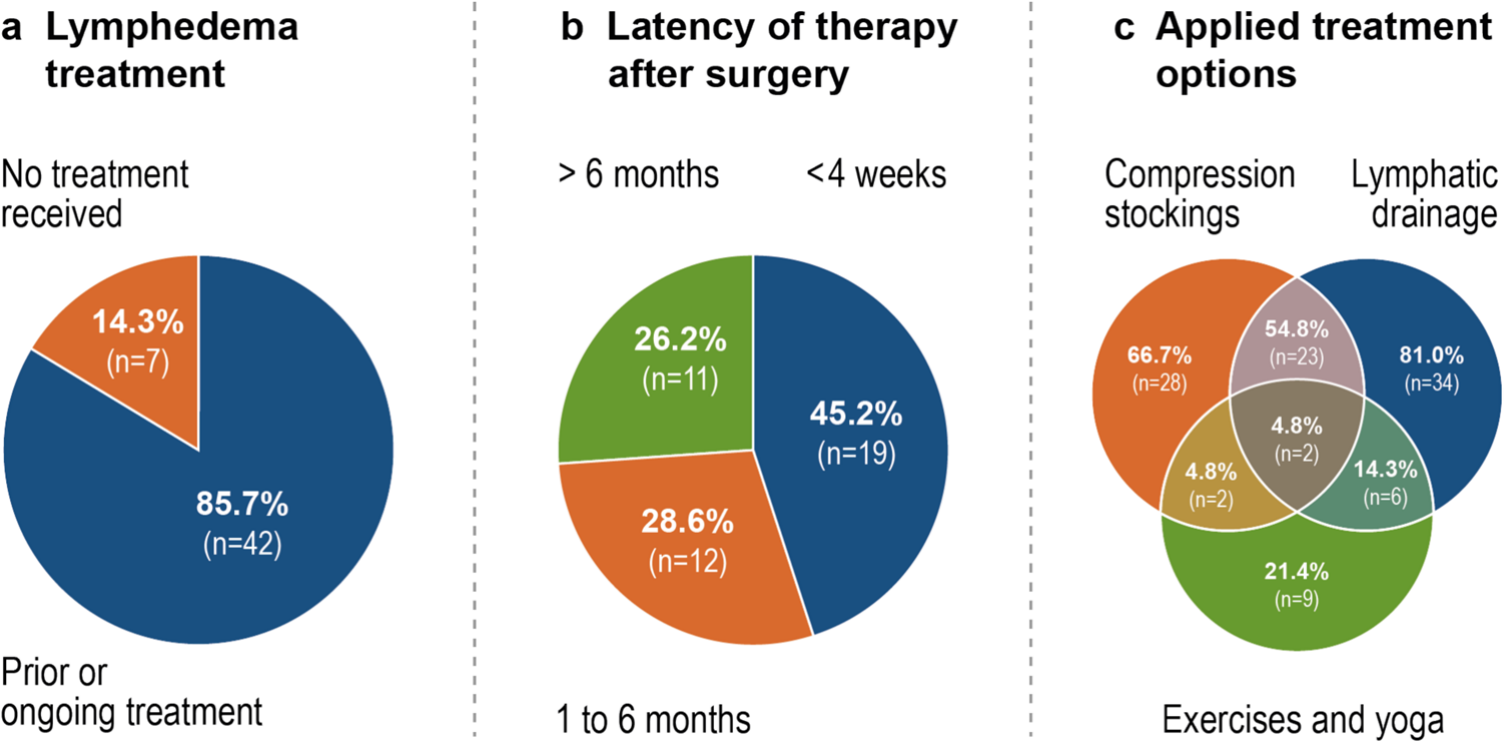
Lymphedema treatment in patients with clinically diagnosed lymphedema (n = 49)

Characteristics of lymphedema treatment are listed in Fig. 4 and supplementary Figure S5. At the time of their aftercare visit, 42 patients (85.7%) with clinically diagnosed lymphedema reported receiving lymphedema treatment. All patients (n = 49) were aware of their lymphedema diagnosis. In 19 patients (45.2%), lymphedema treatment was initiated within the first four weeks after oncological surgery. The most frequently applied treatments were lymphatic drainage in 34 patients (81.0%) and compression therapy in 28 patients (66.7%), while other treatments such as physical exercise or yoga were practiced less frequently (n = 9, 21.4%). Compression therapy was reportedly applied daily in the majority of patients (n = 24, 85.7%). Lymphatic drainage was most frequently utilized once a week (n = 19, 55.9%), closely followed by twice a week (n = 16, 47.1%).

### Limb volume measurement

Total limb volume revealed asymmetric measurements for lower extremities in patients with lymphedema. Larger limb volumes as measured by the circumference measurement method correlated with the clinical diagnosis of lymphedema in the clinically affected extremity. However, this finding did not reach statistical significance (p = 0.192, Fig. 1c). Moreover, there was no significant correlation between limb volume differences and the GCLQ-GER total score (r_s_ = 0.137, p = 0.169, Fig. 1d).

## Discussion

With this study, we introduce a novel version of the GCLQ in German language and demonstrate its excellent ability to diagnose lymphedema in German-speaking gynecologic cancer patients with high sensitivity and specificity. We thereby provide a tool for easy and effective quantitative lymphedema assessment that can be readily implemented in a clinical setting. We found that patients with clinically diagnosed lymphedema had significantly higher total and symptom cluster scores on the GCLQ-GER compared to patients with lymphedema (p < 0.05). The large AUC for the GCLQ in German language (0.874) was comparable to the AUCs of the GCLQ in English (0.952) and Korean (0.868) [20, 21]. As in the English-language validation, two clusters, for swelling and numbness, showed the best AUC among all symptom clusters. In the German version, a GCLQ score of ≥ 4 was identified as the optimal cut-off value for detecting lymphedema in clinical practice. The internal consistency reliability, measured by Cronbach's alpha, was deemed comparable for the GCLQs in German (0.876), English (0.95) and Korean language (0.83). To further validate these findings and establish their generalizability, multicenter studies are needed.

Some of our findings deserve further discussion: Clinically, lymphedema was diagnosed in 49 of 102 patients (48.0%) in this study. This is higher than the prevalence reported in other studies and can be explained by differences between the study cohorts [1–3]. For example, in the GOG-244 (LeG) study, only 17% of all patients had advanced disease (i.e., staged > FIGO I) compared with 52.9% in our cohort [4]. In addition, there are comparatively few patients with endometrial cancer (n = 14, 13.7%) and vulvar cancer (n = 10, 9.8%) in our cohort, and these patients have generally lower rates of lymphedema, especially when treated with sentinel LND [28–30]. Only 6.8% of the patients in our cohort received sentinel LND. On the other hand, all were treated with open surgery and comprehensive LND. Moreover, patients with lymphedema may have attended cancer aftercare more frequently. In this study, a significant association between radicality of LND and lymphedema diagnosis could be demonstrated. The number of removed lymph nodes as part of surgical cancer therapy was identified as an important risk factor in lymphedema pathogenesis (p < 0.001). Although this is consistent with other studies, the finding should be interpreted with caution, as it may be biased by the heterogeneity of different gynecologic cancers and disease stages [31–33].

Using a cut-off value of ≥ 4 on the GCLQ-GER yielded a lymphedema diagnosis in 53 (52.0%) of all 102 patients, respectively, corresponding almost exactly with the clinical lymphedema diagnoses. This high conformity between patient-reported and physician-objectified lymphedema diagnosis supports the increasing relevance of PROMs in detecting lymphedema. In line with our findings, recent studies showed positive effects of collecting PROM data to improve health-related quality of life and patient-physician communication in gynecologic oncology [34–36]. Furthermore, there is a need for validated lymphedema questionnaires in German language to focus more on the patient's perspective [33, 37].

Some studies, however, have questioned the validity of the original GCLQ in English language. Brown et al. demonstrated an overestimation of lymphedema prevalence using the GCLQ, showing a higher frequency of LEL when assessed by GCLQ (27/50, 54%) as compared to circumference measurements by nurses (12/50, 24%) [38]. Hayes et al. reported similar findings [39]. Importantly, the results of our study are consistent with most other lymphedema studies using the GCLQ [20–24]. Nevertheless, patients´ inquiries during the GCLQ-GER survey revealed that some items can be understood for symptoms beyond lymphedema. As such, patients used the GCLQ-GER to report symptoms being related to dermatologic, angiologic or neurologic conditions. To associate patient-reported symptoms specifically with lymphedema, a detailed patient history of previous diseases and interventions is mandatory. In the context of clinical practice, an advantage of the GCLQ is that it can be used for longitudinal monitoring of treatment success. Whether the GCQL-GER might also serve as a tool for early detection of sub-clinical lymphedema (i.e., before obvious swelling is present) and to guide treatment initiation needs to be determined in future trials. The high lymphedema treatment rate of 85.7% (n = 42) and awareness of lymphedema diagnosis in 100% of patients (n = 49) contributed to the notion of low prevalences of undiagnosed lymphedema after gynecologic cancer therapy [40]. The frequent finding of lymphedema requiring treatment within the first 4 weeks after surgery (n = 19, 45.2%), emphasizes the need for clinical vigilance for early post-surgery lymphedema [1, 8]. Real-world data from this study confirmed decongestive therapy encompassing compression stockings and lymph drainage as the standard of care for lymphedema treatment [41–43]. However, the daily utilization of compression stockings (n = 24, 85.7%) underlines the large involvement of lymphedema in the activities of daily life.

There are two important limitations of our study that need to be discussed: First, we followed other studies that have validated the GCLQ by comparing its diagnostic performance against a clinical expert examination as the gold standard for lymphedema diagnosis [20, 21]. Although this may not be the most precise method, other techniques such as bioimpedance spectroscopy or longitudinal recording of limb volume changes (LVC) are not clinically established [44].

Furthermore, PROMs might be the more relevant clinical endpoint, as symptoms should be considered more meaningful than mere changes in leg volume. A combination of physical measurements and GCLQ-GER score, e.g. the assessment of GCLQ-GER and LVC over time, could potentially enhance the detection and longitudinal monitoring of lymphedema in the future [45].

Second, our GCLQ-GER assessment did not take into account the data on lymphedema treatment. Therefore, ongoing treatment might have masked the true extent of a transiently decongested lymphedema, which has to be considered as a confounder of both, LVC and GCLQ-GER scoring.

In conclusion, we validated the GCLQ in German language as a reliable and easy-to-use screening tool for lymphedema assessment in gynecologic cancer patients. The introduction of the GCLQ-GER in the clinical practice of cancer aftercare has the potential to help optimize secondary prevention of lymphedema through early detection and treatment initiation, thereby reducing long-term complications and improving quality of life [37, 46]. In addition, it represents a valuable tool for clinical trials yielding data that can be compared internationally.

## Supplementary Information

Below is the link to the electronic supplementary material.

Supplementary file1 (PDF 308 KB)

## Data Availability

The data that support the findings of this study are available from the corresponding author upon reasonable request.
