# Supplementary material for: Lower extremity lymphedema in patients with gynecologic cancer: Validation of the Gynecologic Cancer Lymphedema Questionnaire (GCLQ) in German language and investigation of lymphedema real-world treatment
Source: Arch Gynecol Obstet. 2024 Dec 23;311(4):1151–62. doi: 10.1007/s00404-024-07886-4 (PMC11985603; doi:10.1007/s00404-024-07886-4)
Supplement: Supplementary file 1 — Supplementary file1 (PDF 308 KB) [file 404_2024_7886_MOESM1_ESM.pdf]

ELECTRONIC SUPPLEMENTARY INFORMATION (SI)

Archives of Gynecology and Obstetrics

Title:
Lower extremity lymphedema in patients with gynecologic cancer: Validation of the Gynecologic Cancer Lymphedema Questionnaire (GCLQ) in German language and investigation of lymphedema real-world treatment

Authors:
Henrike Meyer (1), Andreas Hinz (2), Christiane Weisgerber (1), Adrian Pilny (1), Nadja Dornhöfer (1), Anja Mehnert-Theuerkauf (2), Bahriye Aktas (1), Benjamin Wolf (1, 3)*

(1) Department of Gynecology, University Hospital Leipzig, Liebigstr. 20a, 04103 Leipzig, Germany
(2) Department of Medical Psychology and Medical Sociology, Comprehensive Cancer Center Central Germany (CCCG), University Medical Center Leipzig, Leipzig, Germany
(3) Edwin L. Steele Laboratories, Department of Radiation Oncology, Massachusetts General Hospital, Boston, USA.

***Corresponding author:**
Benjamin Wolf
University Hospital Leipzig
Department of Gynecology
Liebigstraße 20a, 04103 Leipzig
E-Mail: benjamin.wolf@medizin.uni-leipzig.de

S1 German translation of the Gynecologic Cancer Lymphedema Questionnaire (GCLQ-GER)

Die folgenden Fragen beziehen sich auf Ihre Erfahrungen mit Beweglichkeit in den letzten 4 Wochen.			
**GCLQ1**	Sind Sie in der Beweglichkeit Ihrer Hüfte eingeschränkt?	Ja □	Nein □
**GCLQ2**	Haben Sie eine eingeschränkte Beweglichkeit Ihres Knies?	Ja □	Nein □
**GCLQ3**	Haben Sie eine eingeschränkte Beweglichkeit Ihres Knöchels (Sprunggelenk)?	Ja □	Nein □
**GCLQ4**	Haben Sie eine eingeschränkte Beweglichkeit Ihres Fußes?	Ja □	Nein □
**GCLQ5**	Haben Sie eine eingeschränkte Beweglichkeit Ihrer Zehen?	Ja □	Nein □
**GCLQ6**	Fühlen sich Ihr Bein oder Ihr Fuß schwach an?	Ja □	Nein □
Die folgenden Fragen beziehen sich auf Beschwerden, die in Ihren Füßen, Hüften, Leisten oder Ihrem Unterleib in den letzten 4 Wochen aufgetreten sind.			
**GCLQ7**	Haben Sie Druckempfindlichkeit (verstärktes Schmerzempfinden bei Berührung) verspürt?	Ja □	Nein □
**GCLQ8**	Haben Sie eine Schwellung bemerkt?	Ja □	Nein □
**GCLQ9**	Haben Sie eine Schwellung mit Grübchenbildung festgestellt? (Grübchenbildung ist, wenn Sie fest auf Ihre Haut drücken und die Delle lange genug bleibt, um sie zu fühlen, wenn Sie mit dem Finger darüber gleiten.)	Ja □	Nein □
**GCLQ10**	Haben Sie eine Rötung festgestellt?	Ja □	Nein □
**GCLQ11**	Haben Sie eine Blasenbildung festgestellt?	Ja □	Nein □
**GCLQ12**	Haben Sie Festigkeit/ Straffheit festgestellt?	Ja □	Nein □
**GCLQ13**	Haben Sie eine erhöhte Temperatur in Ihrem Bein verspürt?	Ja □	Nein □
**GCLQ14**	Haben Sie ein Schweregefühl verspürt?	Ja □	Nein □
**GCLQ15**	Haben Sie ein Taubheitsgefühl verspürt?	Ja □	Nein □
**GCLQ16**	Haben Sie Steifheit verspürt?	Ja □	Nein □
**GCLQ17**	Haben Sie Schmerzen verspürt?	Ja □	Nein □
**GCLQ18**	Haben Sie eine Schwellung der Hüfte verspürt?	Ja □	Nein □
**GCLQ19**	Hatten Sie Schwellungen in der Leiste verspürt? (Genitalbereich, Schamlippen)	Ja □	Nein □
**GCLQ20**	Haben Sie Flüssigkeitsansammlungen festgestellt?	Ja □	Nein □

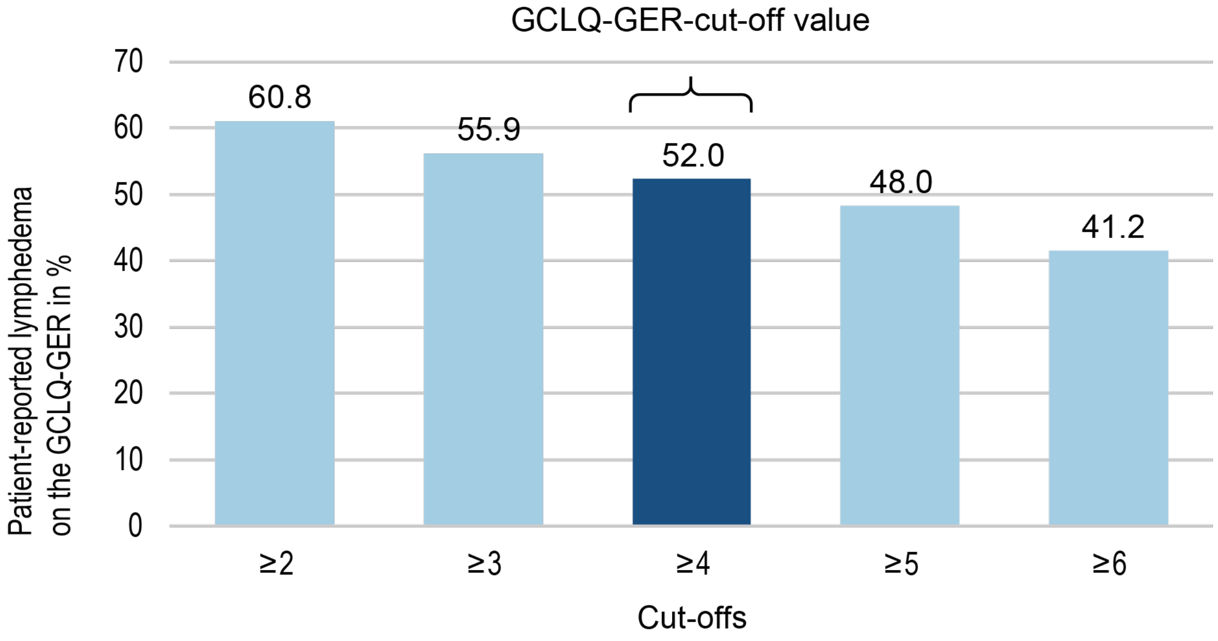

S2 Potential clinical GCLQ-GER-cut-off values for patient-reported lymphedema assessment in clinical practice

S3 Frequency of the patient-reported lymphedema symptoms on the GCLQ-GER

	Item	Cluster	LEL (n=49)	No LEL (n=53)	Total (n=102)
			n (%)	n (%)	n (%)
1	Limited movement of your hip	PF	12 (24.5)	5 (9.4)	17 (16.7)
2	Limited movement of your knee	PF	10 (20.4)	7 (13.2)	17 (16.7)
3	Limited movement of your ankle	PF	7 (14.3)	2 (3.8)	9 (8.8)
4	Limited movement of your foot	PF	10 (20.4)	2 (3.8)	12 (11.8)
5	Limited movement of your toes	PF	11 (22.4)	5 (9.4)	16 (15.7)
6	Leg or foot feels weak	PF	17 (34.7)	7 (13.2)	24 (23.5)
7	Experienced tenderness	N	24 (49.0)	6 (11.3)	30 (29.4)
8	Experienced swelling	SW	38 (77.5)	8 (15.1)	46 (45.1)
9	Experienced swelling with pitting	SW	21 (42.8)	3 (5.7)	24 (23.5)
10	Experienced redness	INF	8 (16.3)	1 (1.9)	9 (8.8)
11	Experienced blistering	INF	1 (2.0)	0 (0.0)	1 (1.0)
12	Experienced firmness/tightness	N	28 (57.1)	5 (9.4)	33 (32.3)
13	Experienced increased temperature in the leg	INF	8 (16.3)	2 (3.8)	10 (9.8)
14	Experienced heaviness	H	28 (57.1)	8 (15.1)	36 (35.3)
15	Experienced numbness	N	28 (57.1)	12 (22.6)	40 (39.2)
16	Experienced stiffness	N	13 (26.5)	2 (3.8)	15 (14.7)
17	Experienced aching	A	28 (57.1)	14 (26.4)	42 (41.2)
18	Experienced hip swelling	LSW	9 (18.4)	0 (0.0)	9 (8.8)
19	Experienced groin swelling	LSW	18 (36.7)	4 (7.5)	22 (21.6)
20	Experienced pockets of fluid	SW	37 (75.5)	3 (5.7)	40 (39.2)

LEL, lower extremity lymphedema; PF, physical function; N, numbness; SW, swelling; INF, infection; H, heaviness; A, aching; LSW, limb swelling

S4 Receiver Operating Characteristics (ROC) for the GCLQ-GER total score and GCLQ-GER symptom cluster

	**Area under the curve (AUC)**	**Asymptotic 95% confidence interval**	
		Lower bound	Upper bound
**GCLQ total score**	0.874	0.802	0.946
**GCLQ symptom cluster**			
(1) Swelling general: Items 8, 9, 20	0.876	0.802	0.950
(2) Numbness: Items 7, 12, 15, 16	0.827	0.746	0.909
(3) Heaviness: Item 14	0.710	0.607	0.813
(4) Aching: Item 17	0.654	0.546	0.761
(5) Swelling Limb: Items 18, 19	0.672	0.565	0.778
(6) Infection: Items 10, 11, 13	0.597	0.486	0.708
(7) Physical function: Items 1–6	0.677	0.571	0.782

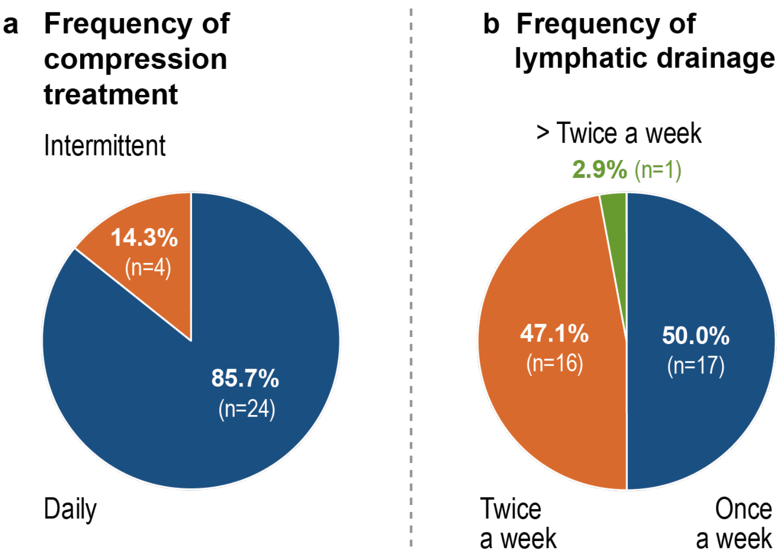

S5 Compression treatment and lymphatic drainage in patients with clinically diagnosed lymphedema (n=49)
